# Application of *in vitro* pollination of opened ovaries to obtain *Brassica oleracea* L. × *B. rapa* L. hybrids

**DOI:** 10.1007/s11627-013-9587-8

**Published:** 2013-12-12

**Authors:** Katarzyna Sosnowska, Teresa Cegielska-Taras

**Affiliations:** Department of Genetics and Breeding of Oilseed Crops, Plant Breeding and Acclimatization Institute - National Research Institute, Strzeszynska 36, 60-479 Poznan, Poland

**Keywords:** *In vitro* pollination, *Brassica oleracea*, *Brassica rapa*, Interspecific hybridization

## Abstract

This study presents the results of experiments concerning: (1) interspecific hybridization of *Brassica oleracea* × *Brassica rapa* via application of *in vitro* placental pollination and (2) embryological analysis of the process of resynthesis of *Brassica napus*. In order to overcome certain stigma/style barriers, *B. rapa* pollen was placed *in vitro* on an opened *B. oleracea* ovary (with style removed). Pollinated ovaries were cultured on Murashige and Skoog (MS) medium. After 24-d culture, the developing embryos were isolated from immature seeds and transferred onto MS medium supplemented with 0.47 μM kinetin, 0.49 μM 1-naphthaleneacetic acid, and 10% (*v*/*v*) coconut water. When the embryos had turned green, they were immediately placed onto MS medium with 100 μM kinetin. After development of the seedling, plantlets were transferred to soil. Chromosome doubling was achieved after another week. Cytometric analysis of nuclear DNA confirmed the hybrid nature of the plants. Resynthesis of *B. napus* can be performed through interspecific hybridization of *B. oleracea* × *B. rapa* followed by embryo rescue and genome doubling.

## Introduction

Currently, increasing the genetic diversity of plants by using *in vitro* culture techniques is a significant challenge in plant breeding programs. Oilseed rape (*Brassica napus* L., AACC, 2*n* = 38) evolved as a natural amphidiploid following spontaneous hybridization between two diploid species (U N 1935), i.e., turnip rape (*Brassica rapa* L., AA, 2*n* = 20) and curly kale (*Brassica oleracea* L., CC, 2*n* = 18). The limited geographical restriction of oilseed rape cultivation, combined with intensive quality breeding (double-low varieties), led to significant limits in the genetic pool of this species. Due to the large number of closely related species of the genus *Brassica*, it is possible to transfer, via distant crossing, new features which are beneficial for oilseed rape breeding material (in terms of improvements in seed yield, disease, and pest resistance, as well as seed quality traits). One way to broaden the range of genetic variation accessible to breeders is to obtain resynthetic (RS) oilseed rape via interspecific crossing between two basic species: *B. oleracea* and *B. rapa* (Rahman 2013).

Resynthesis of *B. napus* has been performed with good results through *in vivo* crossing of *B. rapa* × *B. oleracea*, and then hybrid plants have been obtained through embryo rescue culture (Sosnowska et al. 2010). While *B. oleracea* has been chosen as the maternal parent for crossing with *B. rapa*, sometimes it has been problematic (Lu et al. 2001).

In hybridization between distant species, incompatibility barriers (pre- and postzygotic obstacles) often prevent interspecific crosses. In prezygotic barriers, pollen tubes may start to grow, but then they do not reach the ovules due to the presence of incompatibility mechanisms operating in the stylar tissue. Furthermore, postzygotic barriers may lead to a restriction of embryo growth and development, due to a lack of endosperm development (Zenkteler 1990).

Direct *in vitro* pollination of stigma or pistils and opened ovaries or ovules may be useful in overcoming incompatibility barriers (Zenkteler 1991, 2000). The best results have been achieved in species with large ovaries containing many ovules, such as those belonging to Brassicaceae, Caryophyllaceae, Papaveraceae, Primulaceae, and Solanaceae families (Rangaswamy 1977; Zenkteler 1999). For example, Kameya and Hinata (1970) used ovule pollination to obtain hybrids between *Brassica chinensis* and *Brassica pekiniensis*.

Successful application of this method largely depends on the age of the embryo being rescued and cultured *in vitro*. Hybrid plants have been relatively easily developed from embryos at the autotrophic phase of growth, which begins at the heart stage (Zenkteler 1990).

The aim of this study was to elaborate a method for the interspecific crossing of *B. oleracea* × *B. rapa* through *in vitro* pollination of the opened ovary and thus obtain a new genotype (resynthesis) of *B. napus.*

## Materials and Methods

### *Plant material.*

In the experiment on the interspecific cross, *B. oleracea* L. ssp. *acephala* var. *sabellica* (curly kale) cv. ‘Vitessa’ was used as the female parent, while *B. rapa* L. ssp. *rapifera* (turnip rape), spring type cvs. ‘Kova’ and ‘Skye’ and winter type cvs. ‘Credit’, ‘Ludowy’, and ‘Premium’ were selected as pollen donors. Parental plants were grown in growth cabinets. Closed flower buds were harvested from curly kale about 48 h before opening. These flower buds were surface-sterilized (in a laminar air-flow cabinet) for 20 s in 70% (*v*/*v*) ethanol followed by 3 min in chlorine water and rinsed two times in sterile water.

### *Embryo rescue.*

For *in vitro* culture conditions, the petals and some sepals were removed and the stigma was cut off jointly with the style, and the upper part of the ovary was cut open to expose the ovules. Pollen grains from the anthers of flowers (of turnip rape) were placed directly on the surface of the exposed ovules (curly kale). These pollinated ovaries were transferred to culture medium in glass tubes (30 mL capacity containing 6 mL culture medium; four to five ovaries placed per glass tube). Tubes were plugged with cotton wool. Murashige and Skoog (MS) medium (Murashige and Skoog 1962) was supplemented with 2% (*w*/*v*) sucrose and the pH was adjusted to 5.6 before autoclaving. Explants were incubated at 20–22°C over a 16-h photoperiod.

Enlarged ovules were isolated from the ovary 14 d after *in vitro* pollination and transferred to MS medium supplemented with 0.47 μM kinetin and 0.49 μM 1-naphthaleneacetic acid (NAA), 10% (*v*/*v*) coconut water, and 2% (*w*/*v*) sucrose. Then, after a further 10 d, embryos from the enlarged ovules were dissected and transferred to the same, fresh medium. When the explants had turned green, they were transferred to MS medium with 100 μM kinetin. The shoots were rooted on MS medium with 49.2 μM indole-3-butyric acid (IBA) (Cegielska-Taras et al. 2002). Plantlets were transferred to soil.

### *Cytometric analysis.*

Young leaf tissue (∼1 cm^2^) was harvested from plants growing in the growth cabinets and used for flow cytometry. Samples were prepared according to Galbraith et al. (1983) with some modifications. Plant tissue was chopped with razor blade in a Petri dish, containing 2 mL lysis buffer, with addition of 5.71 μM 4′,6-diamidino-2-phenylindole and 15 × 10^3^ μM β-mercaptoethanol. Suspensions were passed through a nylon filter with 30-μm mesh size and the analyses were performed using PAII (Partec, Germany) flow cytometer. For each leaf sample, 5,000–8,000 nuclei were analyzed with five replications, using a logarithmic scale. Histograms were analyzed with the use of a DPAC v. 2.2 software (Partec Gmbh, Germany).

### *Chromosome doubling.*

Plants at the four- to six-leaf stages were vernalized at 4°C for 7 wk. Subsequently, the number of chromosomes in the hybrid plants was doubled by dipping the roots in 0.05% (*w*/*v*) colchicine solution for 24 h and, after washing the roots, the plants were transferred back into soil. Further development of plant RS oilseed rape occurred in a greenhouse.

### *Embryological study.*

Pollen germination on the upper part of the ovary was observed under a fluorescent microscope using aniline blue (0.1 g L^−1^ H_2_O, pH = 7.2).

The enlarged ovaries and ovules were fixed in FAA (5 mL 40% formalin [*v*/*v*], 5 mL glacial acetic acid, and 90 mL 70% [*v*/*v*] ethanol), passed through alcohol–xylene series (in a graded ethanol series from 70 [*v*/*v*] to 100% [*v*/*v*], 1 h in each) and embedded in Paraplast®. Sections were cut at a 10 μm thickness and stained with Heidenhein’s iron hematoxylin (Merck; 1.5 g L^−1^ of 80% [*v*/*v*] ethanol) and counter-stained with Fast Green FCF (Merck) (0.03 g L^−1^ of clove oil). The material for embryological studies was fixed at various times after pollination. Embryogenesis was observed under a light microscope (Zeiss Axioscope A1, Jena, Germany) and micrographs were recorded using AxioVision software and a multimedia digital camera.

## Results

*B. oleracea* L. ssp. *acephala* var. *sabellica* cv. ‘Vitessa’ and five varieties of spring and winter *B. rapa* L. ssp. *rapifera* were used in interspecific hybridization. During the study, 420 ovaries with exposed ovules were *in vitro* pollinated on MS medium. Several days after pollination (DAP), 217 enlarged ovules were observed. From these, 179 were prepared and cultured *in vitro* for further development, while the remaining 38 were fixed for embryological analysis.

**Figure 1.**
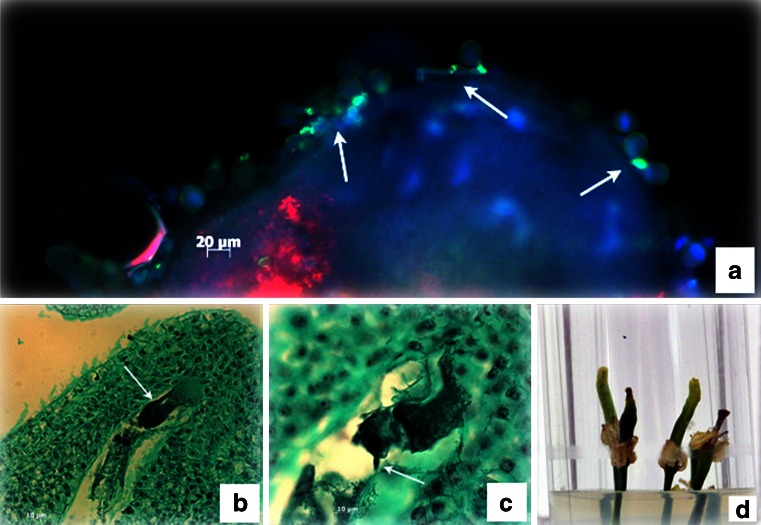
*In vitro* pollination of ovaries. (*a*) Germination of pollen grains of *B. rapa* (*arrows*) on an opened ovary of *B. oleracea*, 24 h after pollination. (*b*) *B. rapa* pollen tube penetrating the *B. oleracea* embryo sac (*arrow*), 48 h after pollination. (*c*) Male *B. rapa* gamete enters the egg cell of *B. oleracea* (*arrow*). (*d*) Enlarged ovaries *B. oleracea* × *B. rapa*, 5 DAP.

Germination of pollen grains on ovules (exposed from the ovary) was observed after 24 h of culture (Fig. 1*a*). Pollen tubes had entered the embryo sac and were visible in many of the analyzed ovules 48 h after *in vitro* pollination (Fig. 1*b*). Embryological slides of ovaries 72 h after pollination showed that male gametes had penetrated the egg cell of the embryo sacs (Fig. 1*c*), and 5 d after pollination some ovaries were enlarged (Fig. 1*d*).

**Figure 2.**
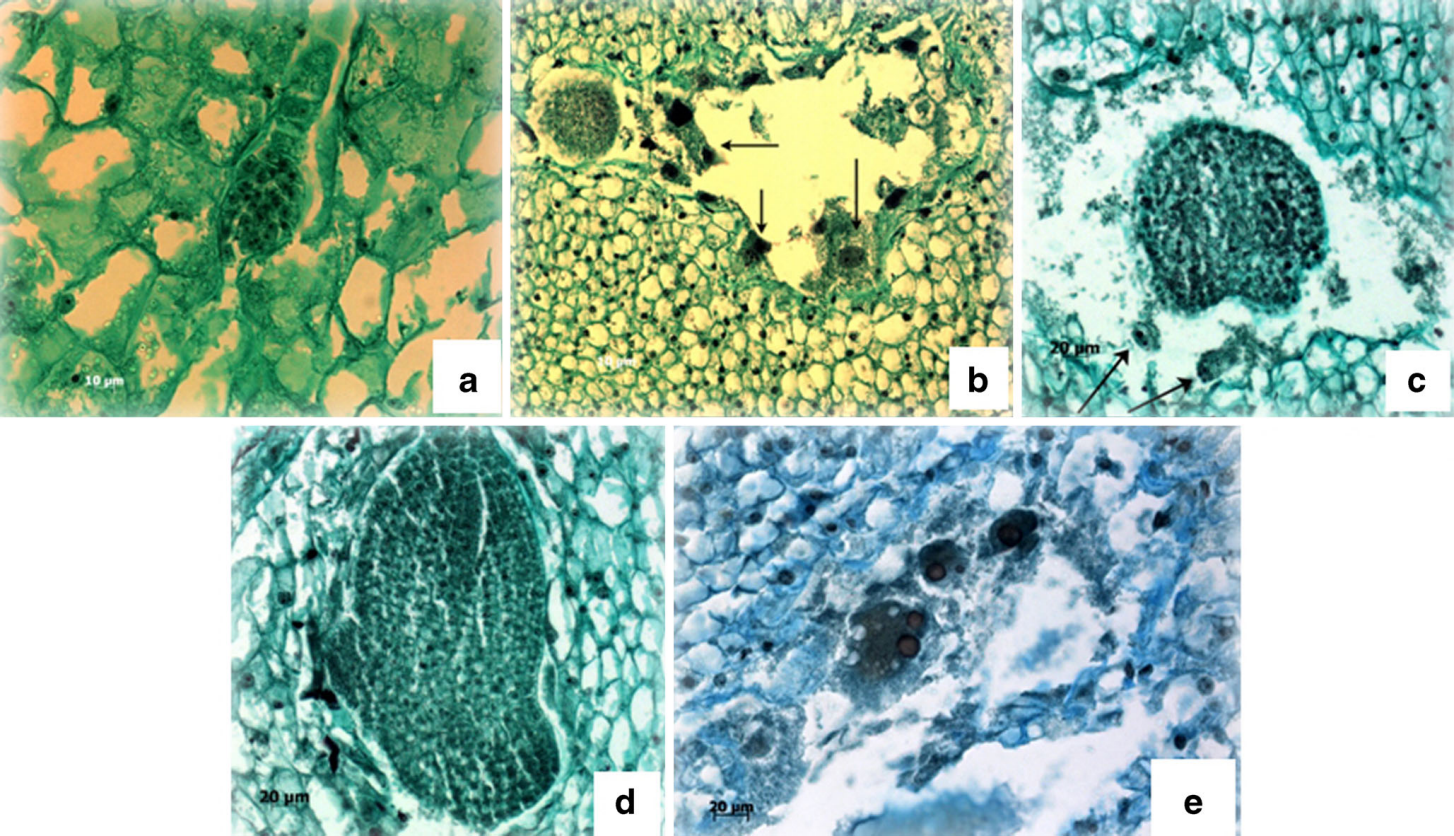
Embryological analysis of enlarged ovules. (*a*) Globular embryo with suspensor of *B. oleracea* × *B. rapa*, endosperm absent, 12 DAP. (*b*) Globular embryo of *B. oleracea* × *B. rapa*, hybrid endosperm (*arrows*), 14 DAP. (*c*) Heart stage embryo of *B. oleracea* × *B. rapa*, residual endosperm (*arrows*), 15 DAP. (*d*) Torpedo stage embryo of *B. oleracea* × *B. rapa*, 17 DAP. (*e*) Hybrid, nuclear endosperm of *B. oleracea* × *B. rapa*, 13 DAP.

**Figure 3.**
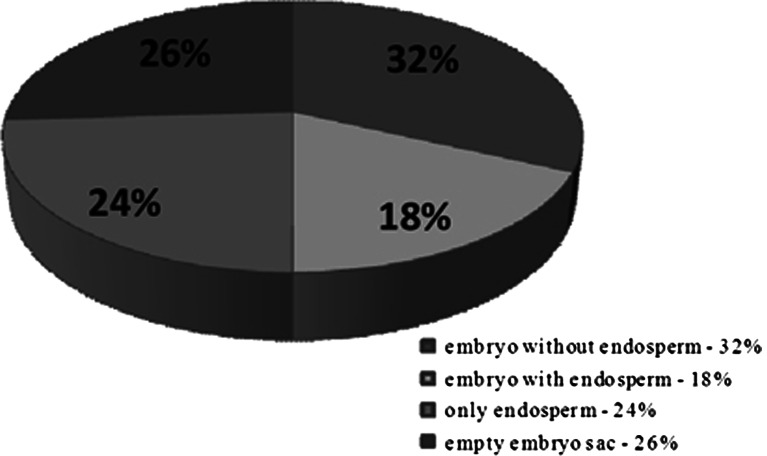
Embryological analysis of enlarged ovules, 12–17 DAP.

Embryological analysis of ovules, 12–17 DAP, revealed the presence of embryos and endosperm at different stages of development (Fig. 2). Among enlarged ovules, some of them contained embryos with endosperm, some with embryos or endosperm only, and others had empty embryo sacs, as shown in Fig. 3. At 12 DAP, globular embryos of various sizes were observed, often with well-developed suspensors (Fig. 2*a*); however, endosperm was often not present. Normally developed endosperm rarely occurred simultaneously with globular embryos 14 DAP (Fig. 2*b*). Endosperm was characterized by various numbers, sizes, and shapes of nuclei and multinucleoli.

Fifteen to 17 d after pollination, early heart- (Fig. 2*c*) and early torpedo-stage embryos (Fig. 2*d*) were identified as well. In some of the enlarged ovules, embryos were not present; however, nuclear endosperm was visible (Fig. 2*e*).

**Figure 4.**
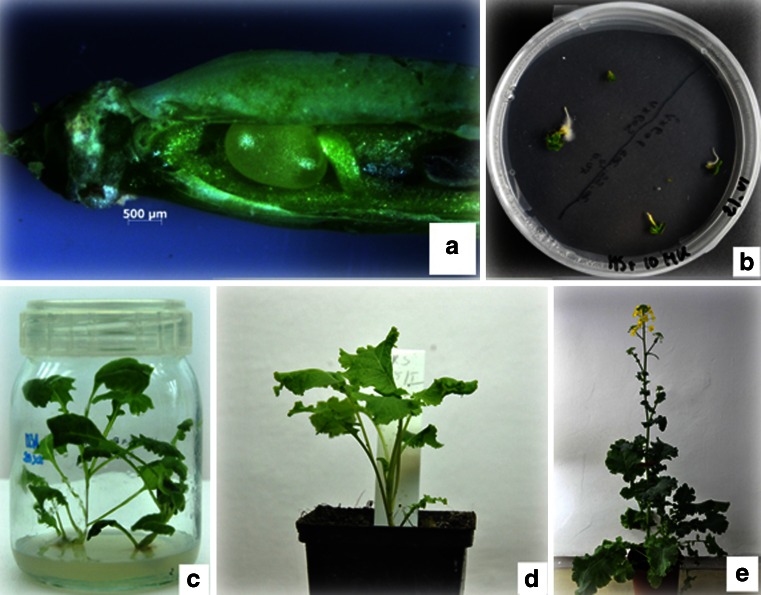
Embryo rescue and plant regeneration. (*a*) Enlarged ovule inside an opened ovary, 14 DAP. (*b*) Mature *B. oleracea* × *B. rapa* embryos germinating on medium. (*c*) Fully formed seedlings with roots. (*d*) Hybrid plants of *B. oleracea* × *B. rapa*. (*e*) Flowering hybrid plant after vernalization.

Embryological observations of *in vitro* pollinated ovules and the development of hybrid embryos allowed a determination of the time at which young embryos must be transferred to medium, due to the failure of endosperm to support further embryo development. Fourteen days after pollination, enlarged ovules (Fig. 4*a*) were isolated and transferred onto MS medium supplemented with NAA, kinetin, and coconut water. After a further 10 d of culture, mature embryos were dissected from the ovules and were also cultured on the same, but fresh medium (Fig. 4*b*). After several weeks, the resultant explants were transferred onto MS medium with 2% (*w*/*v*) sucrose, with the addition of kinetin and IBA, in order to foster the development of shoots and roots (Fig. 4*c*). Afterwards, plantlets were transferred to soil (Fig. 4*d*) and after vernalization, hybrid plants were grown in a greenhouse (Fig. 4*e*).

**Figure 5.**
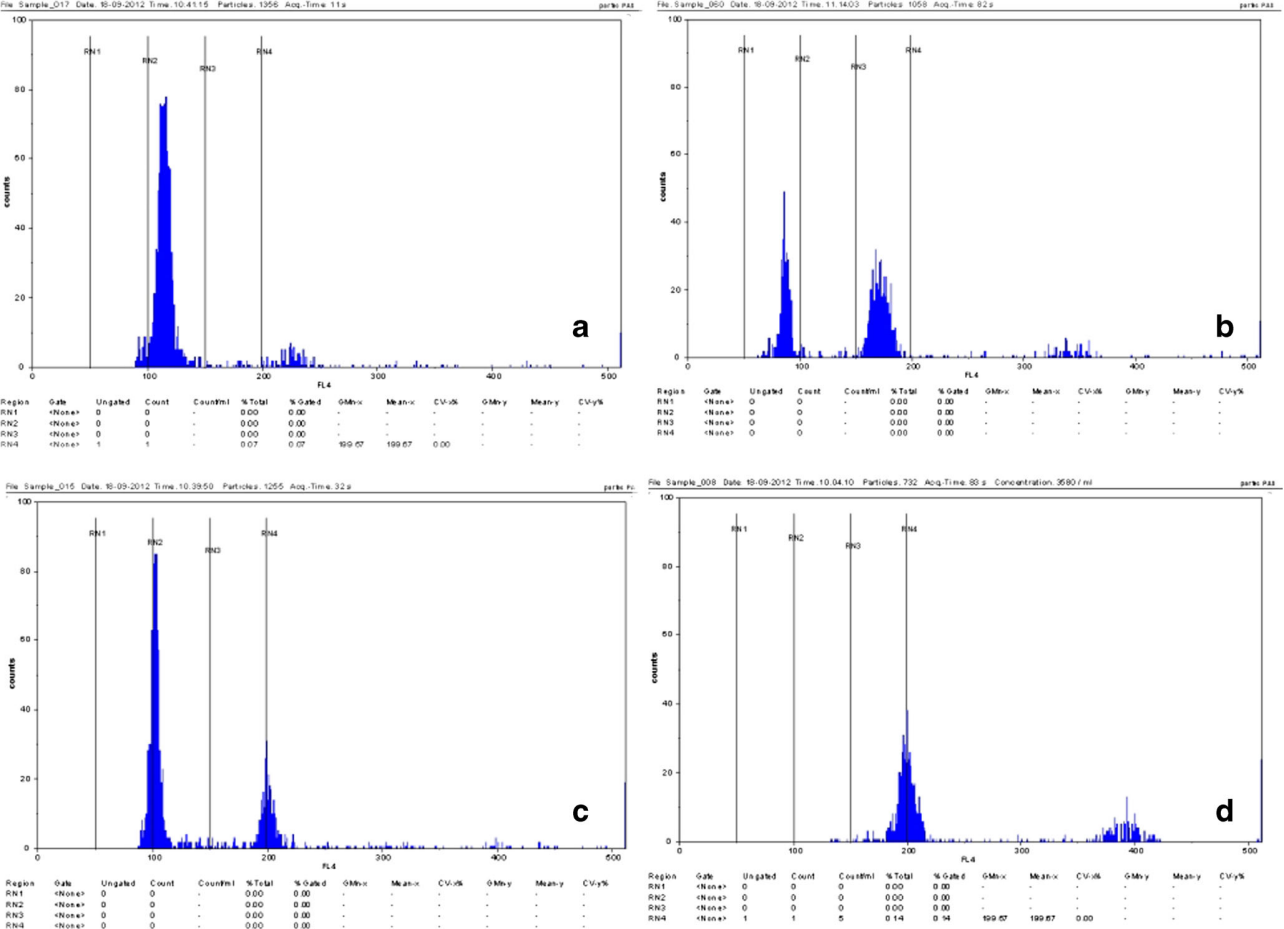
Histograms from flow cytometric analysis showing the ploidy status of (*a*) *B. oleracea*, (*b*) *B. rapa*, and (*c*) resynthesized amphihaploid *B. napus* in comparison with (*d*) normal amphidiploid *B. napus* cv. ‘Monolit’.

The numbers of pollinated ovaries, enlarged ovules, and resultant hybrid plants in different combinations of crosses are presented in Table 1. After *in vitro* pollination of 420 curly kale ovaries with pollen from turnip rape, 179 enlarged ovules were isolated, and 24 plants were recovered. Plants obtained from interspecific crossing were also examined for nuclear DNA content via flow cytometry. Analysis of leaf samples of received putative hybrids showed that they were amphihaploid (*n* = 19) as expected (Fig. 5). These plants were treated with colchicine in order to obtain amphidiploid *B. napus* plants (2*n* = 38).

**Table 1.** Results of interspecific crossing between *B. oleracea* and *B. rapa* via *in vitro* pollination

Cross *B. oleracea* × *B. rapa*	No. of pollinated ovaries	No. of enlarged ovules (A)	No. of hybrid plants (B)	Ratio (B:A)
Curly kale cv. ‘Vitessa’ × turnip rape cv. ‘Kova’^a^	70	19	3	0.158
Curly kale cv. ‘Vitessa’ × turnip rape cv. ‘Skye’^a^	48	15	5	0.333
Curly kale cv. ‘Vitessa’ × turnip rape cv. ‘Credit’	64	19	2	0.105
Curly kale cv. ‘Vitessa’ × turnip rape cv. ‘Ludowy’	151	59	5	0.085
Curly kale cv. ‘Vitessa’ × turnip rape cv. ’Premium’	87	67	9	0.134
Total	420	179	24	0.134

^a^Spring variety

The average efficiency of obtaining new oilseed rape plants from enlarged ovules of curly kale crossed with different spring and winter varieties of turnip rape was around 13.4%. With regard to the resultant hybrid plants, there was a negligible difference between the spring and winter type of turnip rape in the crosses performed.

## Discussion

There are certain interspecific hybrids of *B. oleracea* and *B. rapa* that are very difficult to obtain via conventional sexual crossing (Chen and Heneen 1989; Lu et al. 2001). Such interspecific hybrids, however, can be obtained by *in vitro* or *in vivo* pollination in conjunction with embryo rescue.

Experiments involving crossing two genetically distant parental species can be very difficult or, in many cases, impossible. It is easier to produce progeny when species with a similar gene pool are crossed. Olsson (1960) observed that crosses within the genus *Brassica* occur most frequently when the maternal parent had a greater number of chromosomes than the paternal one. However, Takeshita et al. (1980) showed that when *B. oleracea* was used as a “mother”, interspecific hybrids were much easier to obtain through the *in vitro* culture of embryos. *B. oleracea*, as a cytoplasmic donor, may also be a novel genetic resource for genes corresponding to quality and other traits to be transferred to *B. napus*.

Prezygotic incompatibility barriers between genetically distant species can be avoided or reduced by applying defined methods of pollination and *in vitro* culture, such as pollination of immature pistils, pollination of pistils with mixtures of compatible (inactivated by radiation or chemicals) and incompatible pollen, pollination of opened ovaries and placenta, or *in vitro* fertilization (Zenkteler et al. 1987; Tuyl and De Jeu 1997). Postzygotic incompatibility barriers, such as degeneration of embryos during embryogenesis, improper development of embryos (very often caused by abnormal development of the endosperm [Olsson 1960; Wojciechowski 1985]), can be also avoided by *in vitro* culturing of either isolated embryos or the maternal flower organs in order to induce further growth (Zenkteler 1990). By using these methods, regeneration of hybrid plants is possible. Species of the genus *Brassica* belong to those that employ a sporophytic incompatibility system, where pollen germination is blocked on the surface of the stigma (Zenkteler et al. 1987).

In the present study, in order to avoid the prezygotic barriers of crossing, opened ovaries (cut at the floral apex) were pollinated *in vitro*. Postzygotic barriers were overcome by *in vitro* culture of enlarged ovules with embryos, which were isolated 14 DAP (embryo rescue technique). After a further 10 d, mature embryos were isolated and cultured. Finally, hybrid plants were obtained which were confirmed by cytometric analysis of DNA content. Flow cytometry is more convenient and rapid compared to the traditional karyotyping methods and the use of other morphological characteristics (e.g., size and number of leaf stomata), especially in the analysis of generated hybrid plants.

Application of these methods makes it possible to obtain *B. napus* hybrid plants from crosses between *B. oleracea* and *B. rapa*. Resynthesized *B. napus* is strategically important for the practical breeding of new oilseed rape to increase the gene pool for this crop.
